# Transcriptomic Profiling Reveals the Antiapoptosis and Antioxidant Stress Effects of *Fos* in Ischemic Stroke

**DOI:** 10.3389/fneur.2021.728984

**Published:** 2021-10-21

**Authors:** Qiancheng Mu, Yuxuan Zhang, Long Gu, Stefan T. Gerner, Xiancheng Qiu, Qianke Tao, Jinwei Pang, Ghosh Dipritu, Lifang Zhang, Shigang Yin, Yong Jiang, Jianhua Peng

**Affiliations:** ^1^Department of Neurosurgery, The Affiliated Hospital of Southwest Medical University, Luzhou, China; ^2^Luzhou Key Laboratory of Neurological Diseases and Brain Function, The Affiliated Hospital of Southwest Medical University, Luzhou, China; ^3^Department of Neurology, University Hospital Erlangen-Nuremberg, Erlangen, Germany; ^4^Academician (Expert) Workstation of Sichuan Province, The Affiliated Hospital of Southwest Medical University, Luzhou, China; ^5^Institute of Epigenetics and Brain Science, Southwest Medical University, Luzhou, China; ^6^Sichuan Clinical Research Center for Neurosurgery, The Affiliated Hospital of Southwest Medical University, Luzhou, China

**Keywords:** hypertension, ischemic stroke, *Fos*, oxygen-glucose deprivation, apoptosis, oxidative stress, mitochondria

## Abstract

Arterial hypertension is considered the most prevalent risk factor for stroke. Both pathophysiologic and clinical data previously acquired suggest a strong correlation between the hemodynamic nature of arterial hypertension and an increase in the risk of ischemic insult to tissues. However, the knowledge of specific molecular interactions between hypertension and ischemic stroke (IS) is limited. In this study, we performed systematic bioinformatics analysis of stroke-prone spontaneous hypertensive brain tissue samples of rats (GSE41452), middle cerebral artery occlusion of brain tissue samples of rats (GSE97537), and peripheral blood array data of IS patients (GSE22255). We identified that *Fo*s, an immediate-early gene (IEG) that responds to alterations in arterial blood pressure, has a strong correlation with the occurrence and prognosis of IS. To further evaluate the potential function of *Fos*, the oxygen–glucose deprivation model and RNA sequencing of HT22 neuronal cells were performed. Consistent with the sequencing results, real-time quantitative PCR and Western blot indicate that *Fos* was elevated at 3 h and returned to normal levels at 6 h after oxygen–glucose deprivation. Knock-down of *Fos* by lentivirus significantly increased the oxidative stress level, neuronal apoptosis, and inhibited the mitochondrial function. In conclusion, *Fos* acts as an important link between hypertension and IS. Furthermore, *Fos* can be used as a potential biomarker for target therapy in the prevention of stroke among hypertensive patients and also potential treatment targeting apoptosis and oxidative stress after its onset.

## Introduction

Ischemic stroke (IS) is still a leading cause of death and disability worldwide, despite a decline in stroke mortality due to improved recognition and management of cardiovascular risk factors (1, 2). Arterial hypertension is a major robust non-modifiable risk factor for cardiovascular disease (CVD), particularly cerebrovascular events (3). The cross-linking of stroke and hemodynamics makes the blood pressure management of stroke patients challenging, requiring accurate diagnosis and precise definition of treatment goals. There are many studies about the genomic regions associated with hypertension, and some genetic characteristics of IS susceptibility among different ethnic groups have been reported (4–6). However, there are no effective biomarkers for screening individuals with high stroke risk.

Based on high-throughput sequencing technology, clinical and basic stroke studies have provided resource data of stroke and revealed the transcriptional regulation mechanisms and potential therapeutic targets of a series of key stroke-related genes (7). In addition, a large number of genes and transcription factors related to hypertension have been revealed in previous studies. Therefore, we questioned whether there may be some common pathophysiological processes and specific key regulator molecules between hypertension and stroke.

In this study, we systematically analyzed the array data of stroke-prone spontaneous hypertensive brain tissue of rats, middle cerebral artery occlusion (MCAO) of brain tissue of rats, and peripheral blood of IS patients. Bioinformatics analysis revealed that *Fos* is an important link between hypertension and IS, which provides a new perspective of hypertension and its participation in the development and prognosis of cerebral stroke (For simplification purposes, throughout this paper, *Fos* denotes gene names in rats and cells, *FOS* denotes gene names in humans, and c-Fos denotes proteins in rats and cells). We further demonstrated that *Fos* exerts antiapoptosis and antioxidant stress effects in the HT22 cell oxygen–glucose deprivation (OGD) model and serves as a promising therapeutic target for the prevention and treatment of stroke.

## Materials and Methods

### Data Collection and Processing

The gene expression matrix of the microarray data numbered GSE41452, GSE22255, and GSE97537 were downloaded from the Gene Expression Omnibus (GEO) database (https://www.ncbi.nlm.nih.gov/geo/). The sequencing platform of GSE41452 was Agilent-028282, corresponding to the GEO platform number GPL14745 (8). The microarray samples of GSE41452 included the Kyoto Wistar–Kyoto rats (WKY) control group, spontaneously hypertensive rats, and hypertension stroke-prone rats at two time points (3 and 6 weeks old; each group has three biological repeats). The platform annotation file of GPL14745 was downloaded by the R software package, which id named GEOquery (version 2.58.0). The GSE41452 expression matrix was normalized by microarray probe transformation, gene name transformation, and log transformation. The maximum expression value taken from different probes of the same gene was used for further analysis. Limma package (version 3.46.0) in *R* software was used to screen out IS-related genes after probe and gene name transformation in GSE97537 brain tissue gene expression data. GSE22255 is the HG-U133_Plus_2 chip platform sample, GPL number GPL570, which contains 40 peripheral blood samples including 20 IS patients and 20 sex- and age-matched controls. The detailed information of the controls and patients is shown in Supplementary Table 2 (9). GSE97537 was generated on GPL1355 (Rat230_2) Affymetrix Rat Genome 230 2.0 Array platform. A total of seven brain samples from MCAO rats and five brain samples from sham-operated rats were included in the GSE97537 dataset.

### Construction of the Weighted Gene Coexpression Network and Screening the Key Module

The order is based on the median value of each gene expressed, and the top 5,000 genes in the GSE41452 expression matrix were selected using the R language for cluster analysis to identify outlier samples and to calculate the Pearson coefficient between any two genes. The pickSoftThreshold function of the *Constructing of the Weighted Gene Co-expression Network* package was applied for the expression matrix. By calculating the scale-free topological fit index of several different soft threshold β (*R*^2^ = 0.9), the appropriate soft threshold β was provided for the construction of the adjacency matrix representing the connection strength among genes (10). A new relation matrix was obtained by transforming the adjacency matrix into a topological overlap matrix (TOM), and the transformation was used to reduce noise and false correlation by calculation of the dissimilarity degree of genes. Genes were hierarchically clustered based on the dissimilarity degree obtained by topological overlap, and dynamic sharing algorithm to cluster the dissimilarity degree to get different gene modules. The Pearson coefficients of genes and sample characteristics in different modules were calculated, and the correlation between modules and sample traits was defined as the average correlation of the containing genes, finally to obtain the module, which is the most related to the sample traits.

### Screening of Key Nodes in the Protein Interaction Network in Key Modules

All the genes in the key modules were inputted into the STRING protein interaction database (https://string-db.org/) to construct the protein–protein interaction (PPI) network with the Cytoscape software (https://cytoscape.org/; version 3.6.1) (11). The cytoHubba plug-in in Cytoscape was used to screen the key nodes of the protein interaction network, and the top 10 nodes were selected as key nodes (genes) according to the degree ranking score (12).

### Cell Culture

HT22 cells were maintained in Dulbecco's modified Eagle's medium (DMEM) basic (Gbico, USA) supplemented with 10% fetal bovine serum (Biological Industries) and a 1% penicillin and streptomycin combination under the condition of 37°C and 5% CO_2_. Cells were harvested by trypsin-EDTA treatment and then seeded in the appropriate dish at a density of 1 × 10^5^ cells/cm^2^ for 24 h (24H) before the assay.

### Oxygen–Glucose Deprivation

HT22 cells were subjected to an OGD state for 3 and 6 h to mimic transient focal ischemic stroke *in vitro*. Serum-free DMEM without glucose (Solarbio, China) was used for OGD. Briefly, cells were cultured in the normal incubator for 24H, then the DMEM basic medium was removed and washed using PBS three times. The serum-free DMEM without glucose was added, and cells were cultured in a hypoxia incubator (Eppendorf, Germany) for 3 and 6 h. Cells obtained were then used for further experiments.

### Total RNA-seq Library Generation and Sequencing

Total RNA was extracted from OGD-treated cells and control cells by using the RNeasy kit (TIANGEN, China). Library preparation was performed by using VAHTS^®^ Universal V6 RNA-seq Library Prep Kit for Illumina (Vazyme, NR604-01/02, China) according to the instructions of the manufacturer. All libraries were sequenced using the Illumina HiSeq X 10 with 150PE reads according to the instructions of the manufacturer. Differentially expressed mRNAs were selected for Gene Ontology (GO) and Kyoto Encyclopedia of Genes and Genomes (KEGG) pathway analysis as previously described (13). GO analysis covers three domains: cellular component (CC), molecular function (MF), and biological process (BP) (http://www.geneontology.org). The significance of the KEGG pathways (http://www.genome.jp/kegg) among differentially expressed genes was denoted by the *p*-value. A value of *p* < 0.05 is recommended.

### Real-Time Quantitative PCR

HT22 cells were removed from a Petri dish and were collected for total RNA extraction using an RNeasy kit (TIANGEN, China). Eight hundred nanograms of RNA was used for cDNA synthesis. The RT-qPCR assay was performed according to the detailed procedure described by Ratchford et al. (14). Each reaction was run in triplicate and consisted of 10 ng of cDNA, 16 μl of Power SYBR Green PCR System (Vazyme, China), and 4 mM forward/reverse primers. The fold change in gene expression was calculated using the ^ΔΔ^Ct method (15) with the housekeeping gene, glyceraldehyde-3-phosphate dehydrogenase (GAPDH), as the internal control. The primer sequences are listed below:

*Fos*-forward: GGGAATGGTGAAGACCGTGTCA;
*Fos*-reverse: GCAGCCATCTTATTCCGTTCCC;
GAPDH-forward: ATTGTCAGCAATGCATCCTG;
GAPDH-reverse: ATGGACTGTGGTCATGAGCC.

### Cell Viability Assay

Cell viability was quantified by using an MTT assay kit (Beyotime, China). HT22 cells were seeded onto 96-well plates (1 × 10^4^/well) and cultured in a 5% CO_2_ incubator for 24H. The OGD-treated cells and control cells were added at 10 μl/well of MTT (5 mg/ml) [3-(4,5-dimethyl-2-thiazolyl)-2,5-diphenyl-2H-tetrazolium bromide (MTT)], and subsequently incubated in a 37°C incubator for 4H to form MTT formazan. MTT formazan was solubilized by the addition of formazan solvent (100 μl/well) in a 37°C incubator for 4H. The yield of the MTT formazan product was determined by measuring the absorbance at 570 nm on a microplate reader (OMEGA, Switzerland). By setting the wells with only medium but no cells as the blank control group, data were represented as the percentage of viable cells compared with vehicle-treated control cells, which were arbitrarily assigned a viability value of 100%.

### Western Blot

Cells were homogenized in a RIPA Lysis Buffer (Beyotime, China) containing 20 μl/ml of protease and phosphatase inhibitor cocktail (Beyotime, China). Protein samples were boiled in SDS-polyacrylamide gradient gels (PAGE) loading buffer and subjected to SDS-PAGE. Equal concentrations of solubilized proteins were separated in 12% PAGE and transferred onto polyvinylidene difluoride (PVDF) membranes. Non-specific binding to the membranes was prevented by the incubation with 5% Blotto (5% non-fat dry milk and 1% Tween-20 in TBS) for at least 1 h at room temperature (RT). Membranes were then incubated with various primary antibodies overnight at 4°C. On the following day, membranes were washed and further incubated for 1 h in the presence of horseradish peroxidase (HRP)-conjugated goat anti-rabbit or goat anti-mouse secondary antibodies at room temperature. Clarity Western ECL Substrate A and Peroxide Solution B (Vazyme, China) were used for protein band visualization, and Western blot (WB) exposures were captured using the ChemiDoc XRS + imaging system (VLBER, France). Protein loading was normalized with β-actin (1:8,000, 20536-1-AP, Proteintech, China). Primary antibodies included c-Fos (1:1,000, 2250S, Cell Signaling Technology, USA), SOD2 (1:2,000, 24127-1-AP, Proteintech, China), Pgc-1α (1:800, 2178S, Cell Signaling Technology, USA), Tfam (1:2,000, 22586-1-AP, Proteintech, China), Bcl-2 (1:600, 12789-1-AP, Proteintech, China), and Bax (1:2,000, ab32503, Abcam, UK).

### MDA/GPx/ATP Content

The MDA content was determined using a Lipid Peroxidation MDA Assay Kit (S0131S, Beyotime, China). The GPx content was detected using the Total Glutathione Peroxidase Assay Kit with NADPH (S0058, Beyotime, China), and ATP content was detected using the ATP Assay Kit (S0026, Beyotime, China). All operational processes followed the instructions of the manufacturer.

### Cell Immunofluorescence

HT22 cells, 1 × 10^5^, were seeded onto a polylysine-coated cell climbing slice and cultured for 24H, then treated with OGD insult for 0, 3, and 6 h. Next, the cells were then incubated with a Mito Tracker Red solution (1:5,000, M7512, Invitrogen, USA) for 30 min at 37°C, replaced with 500 μl of 4% paraformaldehyde after 30 min, and further incubated at RT for 20 min with gentle shaking. Termination was carried out using 0.1 M glycine/PBS for 15 min at RT and then permeabilized with 0.1% Triton X-100/PBS for 10 min at RT. After blocking with 10% goat serum/PBS, the cells were incubated overnight at 4°C with cytochrome c antibody (1:200,10993-1-AP, Proteintech, China). Then cells were washed three times with PBS and incubated for 1 h at RT with secondary antibodies (1:200, SA00013-2, Proteintch, China), and finally stained with Mounting Medium with DAPI (4′,6- diamidino-2-phenylindole) (ab104139, Abcam, UK). Cell images were subsequently acquired with a microscope (Nikon, Japan).

### *Fos* shRNA Transfection

Lentiviral particles expressing shRNA against *Fos* and the corresponding control sequence were constructed by Genechem (Shanghai Genechem Co., Ltd.). Knocked-down cells (KD) and negative control cells (NC) were generated by transfecting HT22 cells with pre-synthesized *Fos* (or control) shRNA lentiviral particles as per the standard protocol of the manufacturer. At 72-h post-infection, transfected cells emitted a green fluorescence signal, and puromycin (2 μg/ml) was added to the culture medium for selection and further characterization. The target sequences of shRNA of *Fos* were as follows: KD shRNA: ccGTCTCTAGTGCCAACTTTA. NC shRNA: TTCTCCGAACGTGTCACGT.

### TUNEL Assay

The TUNEL assay was done using the TUNEL assay kit obtained from Beyotime (China, C1089). HT22 cells were seeded onto polylysine-coated cell climbing slice and treated with OGD by the same way with cell immunofluorescence. The next operational processes followed the instructions of the manufacturer.

### Statistical Analyses

Data are presented as the mean ± SEM from at least three independent experiments. Statistical analysis and graph presentation were carried out using GraphPad Prism software version 8.0 (GraphPad Software, La Jolla, CA, USA). The significance of differences between groups was assessed by two-tailed unpaired Student's *t*-test or one-way ANOVA. A value of *p* < 0.05 was considered as a significant difference.

## Results

### Construction of WGCNA Network and Identification of Key Modules

Through the gene clustering analysis of the first 5,000 of the average expression amounts of GSE41452 gene expression matrix, we found that the clusters consisted of the experimental design grouping and internal correlation, indicating that this part of the gene can represent the overall gene expression characteristics, and there were no outlier samples (Supplementary Figure 1).

**Figure 1:**
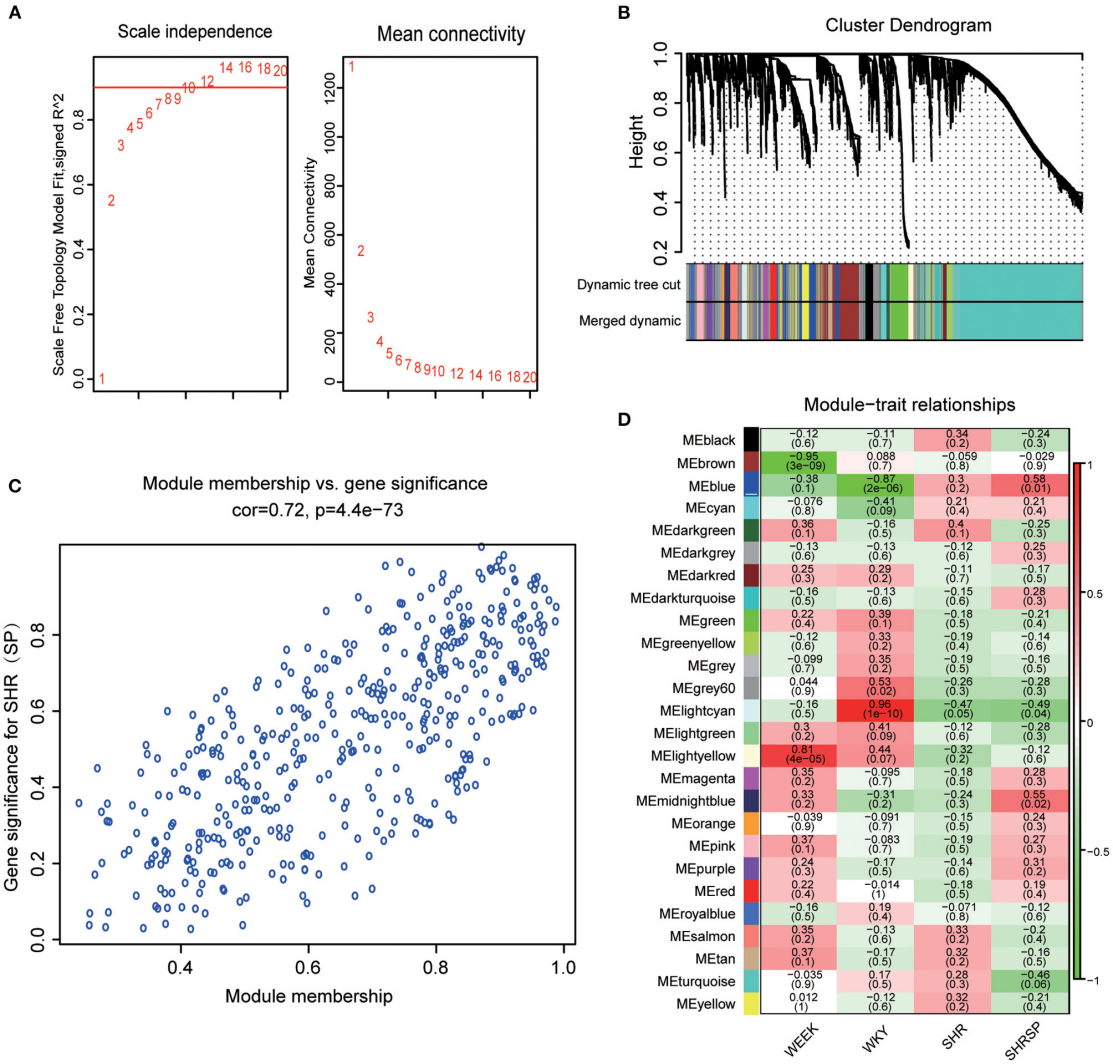
Determination of the best soft threshold and module selection. **(A)** Through the scale independence and mean connectivity to choose the best soft threshold. The red words represent the soft threshold. **(B)** The tree clustering of genes and module form the cluster dendrogram; each color of the module represents a collection of genes with a similar expression. **(C)** Module membership and gene significance were analyzed. **(D)** The ordinate colors represent different modules, the abscissa represents different sample characters, the corresponding heat map shows its Pearson coefficient and probability, and the heat map color represents the correlation (the highest in red and the lowest in blue).

The selection of soft threshold power is an important step in the construction of WGCNA. The network topologies with soft thresholds from 1 to 20 were screened to determine the best soft threshold β for balancing scale independence and mean connectivity. When the scale-free topology fitting index was closest to 0.9, the lowest β value was 8 and was taken as the best soft threshold (Figure 1A). The modules whose dissimilarity degree was <0.25 were set up to merge the other modules (Supplementary Figure 2B), and the dynamic shearing branching algorithm was used to analyze the dissimilarity among the modules, and a total of 26 modules were obtained. Among them, the genes that cannot be included in any screening module were included in the gray module (Figure 1B).

This study also explored the relationship between modules, and the results exhibited a high degree of independence between each module and the phase independence of gene expression in the top 400 genes (Supplementary Figure 2C). To investigate the coexpression similarity of all modules, the eigengene matrix was calculated and clustered according to their correlation (Supplementary Figure 2A). After defining the independence of each module, this study further calculated the relationship between the modules and the sample character. To get the correlation coefficient and probability between the modules and the sample character, we calculated the average Pearson correlation coefficient between the eigengene and the character of the modules. We found that the blue module was highly negatively correlated with WKY traits (*r* = −0.87, *p* = 2 × 10^−6^) and positively correlated with hypertension-related phenotypes with SHRSP traits as the main component (Figure 1D). In addition, we also evaluate not only the correlation between a single gene and the whole module but also the correlation between modules and characters in the blue module (Figure 1C) and found that there was a linear correlation between a single gene and the whole module and also between the module and character (Pearson's correlation coefficient was 0.72, *p* = 4.4 × 10^−73^). The genes of the blue module can represent the correlation between modules and traits. Therefore, the blue module was selected as the key module, and its genes may play an important role in the process of hypertension and stroke.

### Construction of Protein–Protein Interaction in Key Modules and Selection of Key Genes Related to Stroke Process

**Figure 2:**
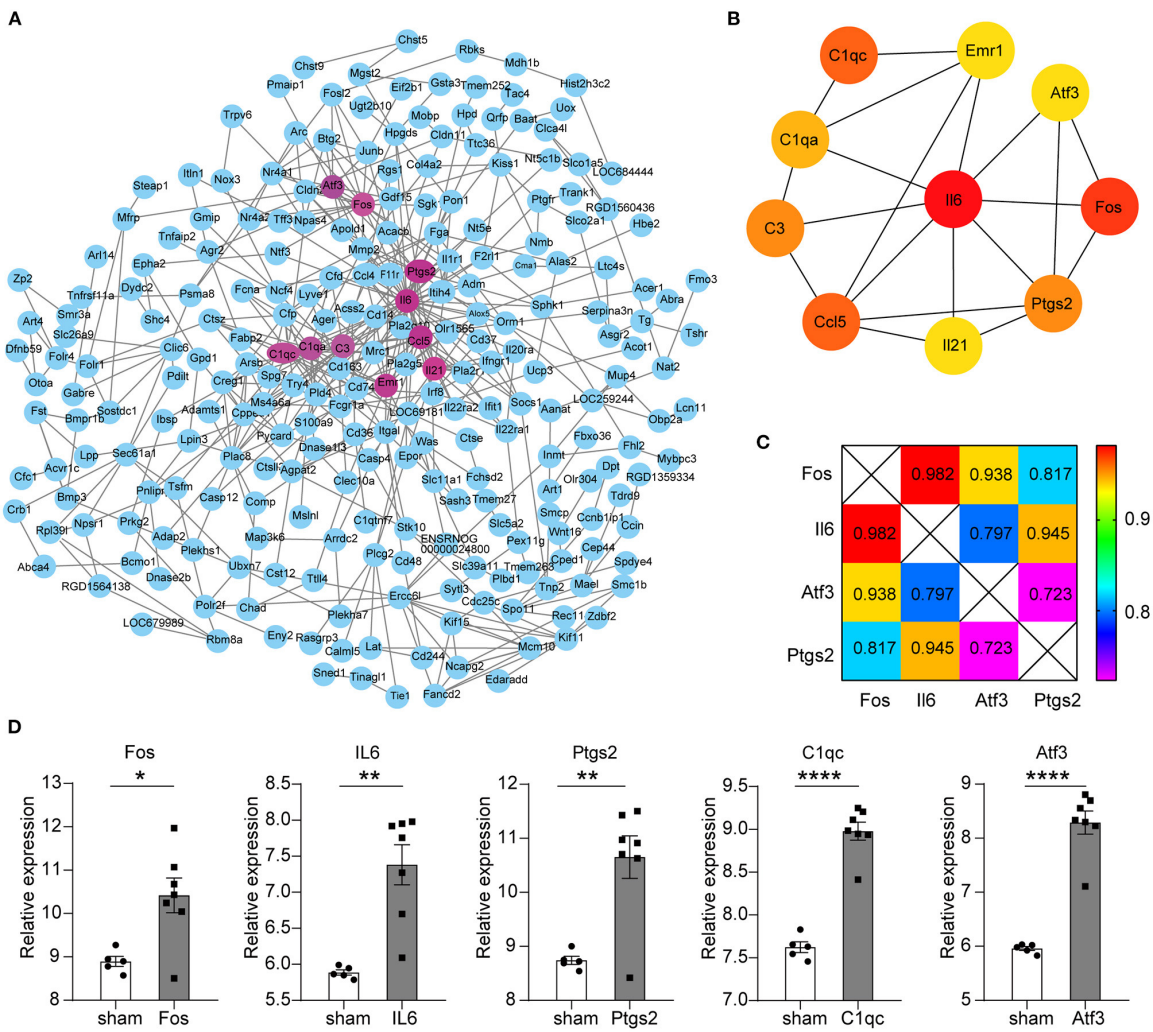
Protein–protein interaction (PPI) network construction for key modules and screening of key genes. **(A)** The circle represents the protein corresponding to the gene, and the wiring represents the interaction. The circles with mauve represent the top 10 key genes in the degree scores. **(B)** Circles represent genes, lines represent interactions, and color represents degree scores, of which red was the highest score. **(C)** The number is the PPI interaction score of differential genes; the different colors indicate different scores. Total score: *Fos* = 2.737, *Il6* = 2.724, *Atf3* = 2.458, *Ptgs2* = 2.485. **(D)** Five differential gene expression levels in GSE97537. The abscissa represents different groups; the ordinate shows the relative gene expression levels, and the error bar is the mean ± SEM, ^*^p < 0.05, ^**^p < 0.01, ^****^p < 0.0001.

All the 450 genes in the key modules were entered into the STRING database for protein interaction analysis. The genes that could not connect any node were removed (Figure 2A). Next, we used the cytoHubba (ver 0.1 version) plug-in to obtain the top 10 key genes in the degree scores; the top 10 genes are *Il6, Fos, Ccl5, C1qc, C3, Il21, Ptgs2, C1qa, Atf3*, and *Emr1* (Figure 2B).

Despite the 450 genes that are closely related to the development of hypertension to stroke-prone in rats, whether these genes have expression changes during the stroke and continue to have an impact from stroke-prone to stroke needs further research. Therefore, brain tissue microarray data of rats with IS were selected to make further analysis to determine whether there is a relationship between the stroke-prone genes and established stroke genes.

GSE97537 was obtained from Affymetrix Rat Genome 2302.0 Array sequencing platform, including 12 brain tissues derived from 7 middle cerebral artery occlusion models (MCAO) and 5 sham-operated SD rats. The limma package (version 3.46.0) was used to analyze the differential expression genes (DEGs) of the annotated expression matrix. Two hundred and seventeen upregulated DEGs and nine downregulated DEGs were obtained according to the screening criteria of |logFC| > 1 and *p* < 0.05. *Il6, Fos, Atf3, Ptg*s2, and *C1qc* are the top part of the DEGs (Figure 2D).

Through further analysis of these five genes, we found that there were close interactions among the four genes except *C1qc* in PPI. We also found that the total combined score of *Fos* was higher than the other three genes through seven evaluation methods, such as Neighborhood in the Genome, Gene Fusion, Co-Occurrence Across Genomes, Co-Expression, Experimental/Biochemical Data, Association in Curated Databases, and Co-Mentioned in PubMed Abstracts (Figure 2C). Therefore, we chose *Fos* as the key gene for further evaluation.

### Expression of *FOS* in the Peripheral Blood of Ischemic Stroke Patients

There were also significant differences in the expression of *FOS* in the peripheral blood of stroke patients. Previously, through the analysis of brain tissue sample data of stroke rats, we found five key genes including *Fos*. Next, we used peripheral blood samples from patients with IS from the open database for verification. Using the IS samples from the gene expression matrix, which had been treated by probe conversion, the *FOS* expression was divided into two groups according to the median of DNA expression, and the DEGs between the two groups were analyzed. The results showed that compared with the control group, many genes were differentially expressed in peripheral blood of IS patients, including 27 genes upregulated and 3 genes downregulated, including *FOS* (Supplementary Table S1).

In our previous analysis, *Fos*/*FOS* was validated in the brain tissues of hypertensive stroke-prone rats, MCAO rats, and peripheral blood of patients with IS. Therefore, *Fos*/*FOS* may play an important role in the transformation of hypertension to stroke and the regulation of gene expression in IS and may be further used as a biomarker and potential therapeutic target for pre-stroke prevention and stroke prognosis.

### RNA-Sequencing Analysis of Oxygen–Glucose Deprivation-Treated HT22 Cells

**Figure 3:**
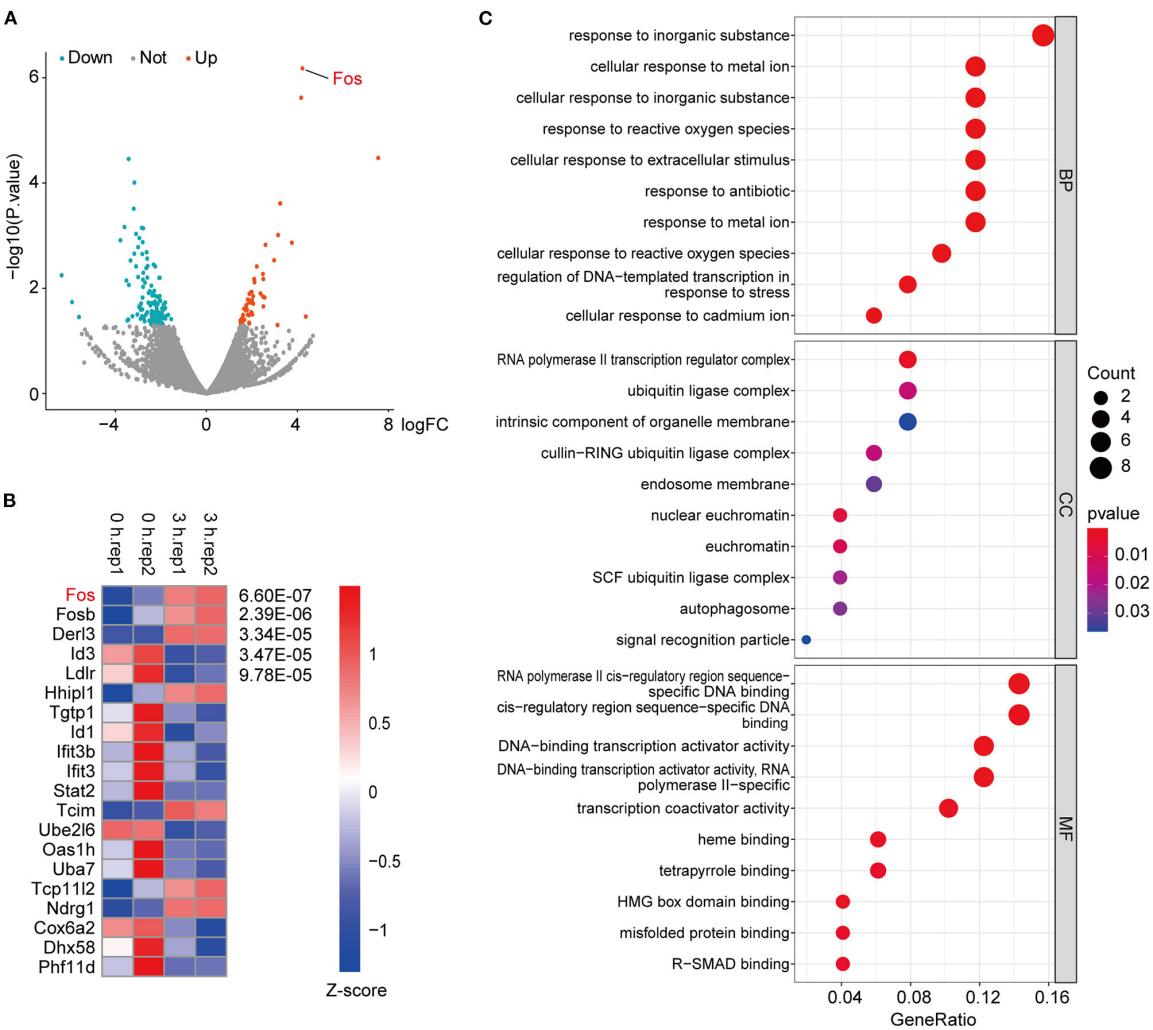
RNA-sequencing of oxygen–glucose deprivation (OGD)-treated HT22 cells. **(A)** These criteria identified 51 upregulated and 119 downregulated genes in 3 vs. 0 h, as shown in the volcano plot, in which the red and green spots represent upregulated and downregulated differentially expressed genes (DEGs), respectively. **(B)** The top 20 genes with the smallest *p*-value among the DEGs were extracted, through normalizing the log2-based TPM + 1 value and the expression matrix by *Z*-score and using the *R* package pheatmap to make a map. **(C)** Gene ontology (GO) enrichment analysis of the upregulated DEGs in RNA-seq data. Gene ratio indicates the number of genes enriched in one pathway compared with the total genes changed in all pathways. Count indicates the number of genes. The colors represent *p*-value, and red is the highest. BP, biological process; CC, cellular component; MF, molecular function.

The samples used in the previous databases were all animal or human organs or tissues; here we used an OGD model of cells. After the OGD state was established for different time points, RNA sequencing was performed. The results showed that *Fos* was significantly upregulated in 3 h compared with 0 h (Figure 3A). In addition, the heatmap of the *p*-value of DEGs among 3 vs. 0 h showed that the *p*-value of *Fos* was the smallest among the DEGs (Figure 3B). By GO analysis of the upregulated DEGs among 3 vs. 0 h, we found that response to inorganic substance was the most significant process, followed by cellular response to metal ion and response to reactive oxygen species (Figure 3C). Therefore, we can conclude that *Fos* may be involved in oxidative stress regulation (16, 17). In the cellular component category, the upregulated DEGs in 3 vs. 0 h were enriched for traits associated with the RNA polymerase II transcription regulator complex, ubiquitin ligase complex, intrinsic component of organelle membrane, and endosome membrane. RNA polymerase II cis-regulatory reign sequence-specific DNA binding was the most abundant in the molecular function category. The results of the GO enrichment revealed that the proteins of DEGs were predominantly binding DNA and have transcriptional regulatory functions, located in the regulator complex.

There were also many DEGs in 6 vs. 3 h and 6 vs. 0 h (Supplementary Figures 3A,B). The upregulated DEGs in both 6 vs. 3 h and 6 vs. 0 h were used for GO analysis and was found that the generation of precursor metabolites and energy is the top process (Supplementary Figures 3C,D). In the cellular component category, the upregulated DEGs in 6 vs. 3 h and 6 vs. 0 h were enriched for traits associated with ribosome, ribosomal subunit, and mitochondrial inner membrane. Structural molecule activity was the most abundant molecular function category. The results of the GO enrichment revealed that the proteins of DEGs in 6 vs. 3 h and 6 vs. 0 h were predominantly binding ribosomes, have structural molecule activity, and are involved in regulating energy generation.

Through KEGG analysis of the RNA-seq data, we found that (1) The KEGG pathway enrichment results of the DEGs in 3 vs. 0 h were mainly enriched for osteoclast differentiation, amphetamine addiction, FoxO signaling pathway, B-cell receptor signaling pathway, and other related signaling pathways, which are mainly involved in regulating cell growth and differentiation, addiction, OGD, and inflammatory immunity pathways (Supplementary Figure 4A); (2) The KEGG pathway enrichment results of the DEGs in 6 vs. 3 h were mainly enriched for ribosome, biosynthesis of amino acids, glycolysis/gluconeogenesis, and hypoxia-inducible factor-1 signaling pathway (HIF-1). These pathways are mainly involved in the synthesis of amino acids and proteins and the cellular response to OGD (Supplementary Figure 4B); (3) The KEGG pathway enrichment results of the DEGs in 6 vs. 0 h and 6 vs. 3 h were generally consistent (Supplementary Figure 4C).

### RT-qPCR and Western Blot Validation

**Figure 4:**
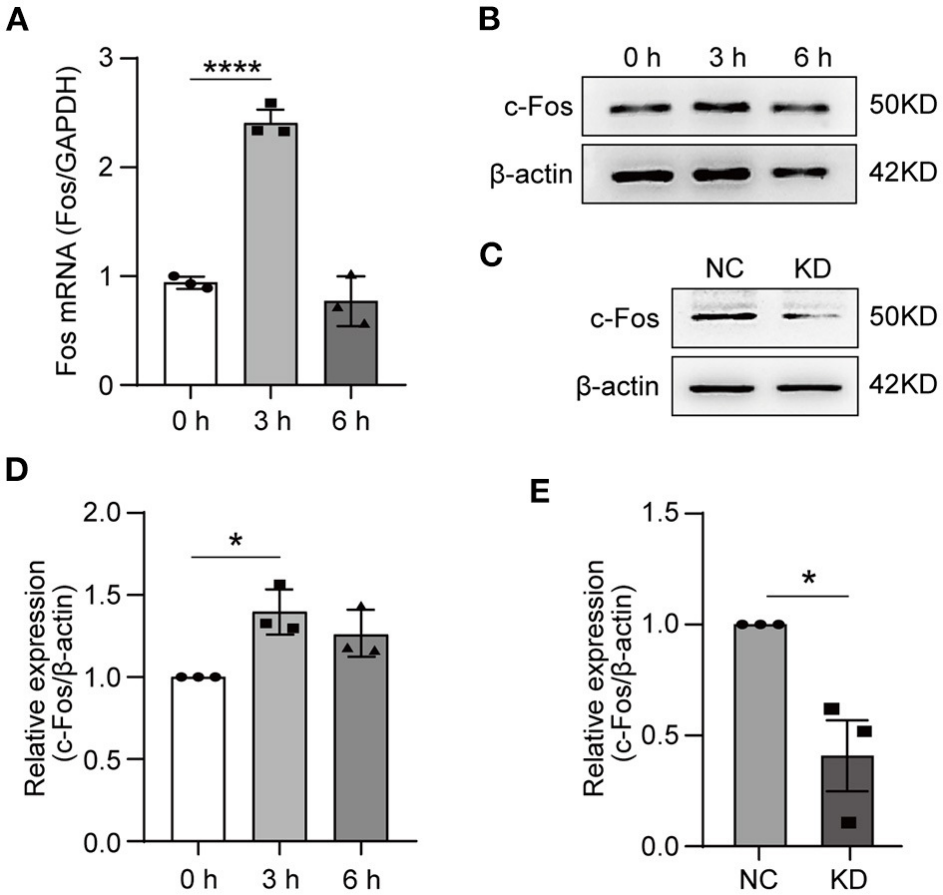
Expression of *Fos* in HT22 cells. HT22 cells were treated by OGD for 0, 3, and 6 h. The total RNA and protein were collected for analysis. **(A)** The *Fos* gene expression level in OGD cells was measured by quantitative real-time PCR. Glyceraldehyde-3-phosphate dehydrogenase (GAPDH) served as the control. **(B)** The c-Fos protein level in OGD cells was detected by Western blot (WB). **(C)** The knockdown efficiency was determined by WB. **(D)** Relative expression levels of c-Fos at each time point after OGD. **(E)** Relative expression levels of c-Fos in the NC group and KD. Each result is expressed as the mean of three independent experiments ± SEM. The statistical analysis of A were performed using one-way ANOVA, and the unpaired *t*-tests were used in **(C)**. ^*^p < 0.05, ^****^p < 0.0001. NC, negative control cells; KD, knock-down cells.

To validate the RNA-seq data, the HT22 cells were treated with OGD for 3 or 6 h. We used RT-qPCR to measure the *Fos* gene expression level after OGD treatment, the results indicated that the expressing level of *Fos* was the highest at 3 h and was not statistically significant at 6 h compared with 0 h (Figure 4A). The WB technique was further used to measure the *Fos* protein level after OGD (Figure 4B). Analysis of WB bands reveals similar results and trends with the RT-qPCR results (Figure 4D). To conclude, we found that *Fos* expression level increased at 3 h and decreased near the normal level at 6 h.

To further solidify our findings, we knocked down *Fos* by lentiviral transfection in HT22 cells. The knocked-down efficiency of *Fos* was confirmed by WB (Figures 4C,E). Ultimately, NC and KD were used for subsequent studies.

### Knockdown of *Fos* Aggravated Neuronal Oxidative Stress After Oxygen–Glucose Deprivation

**Figure 5:**
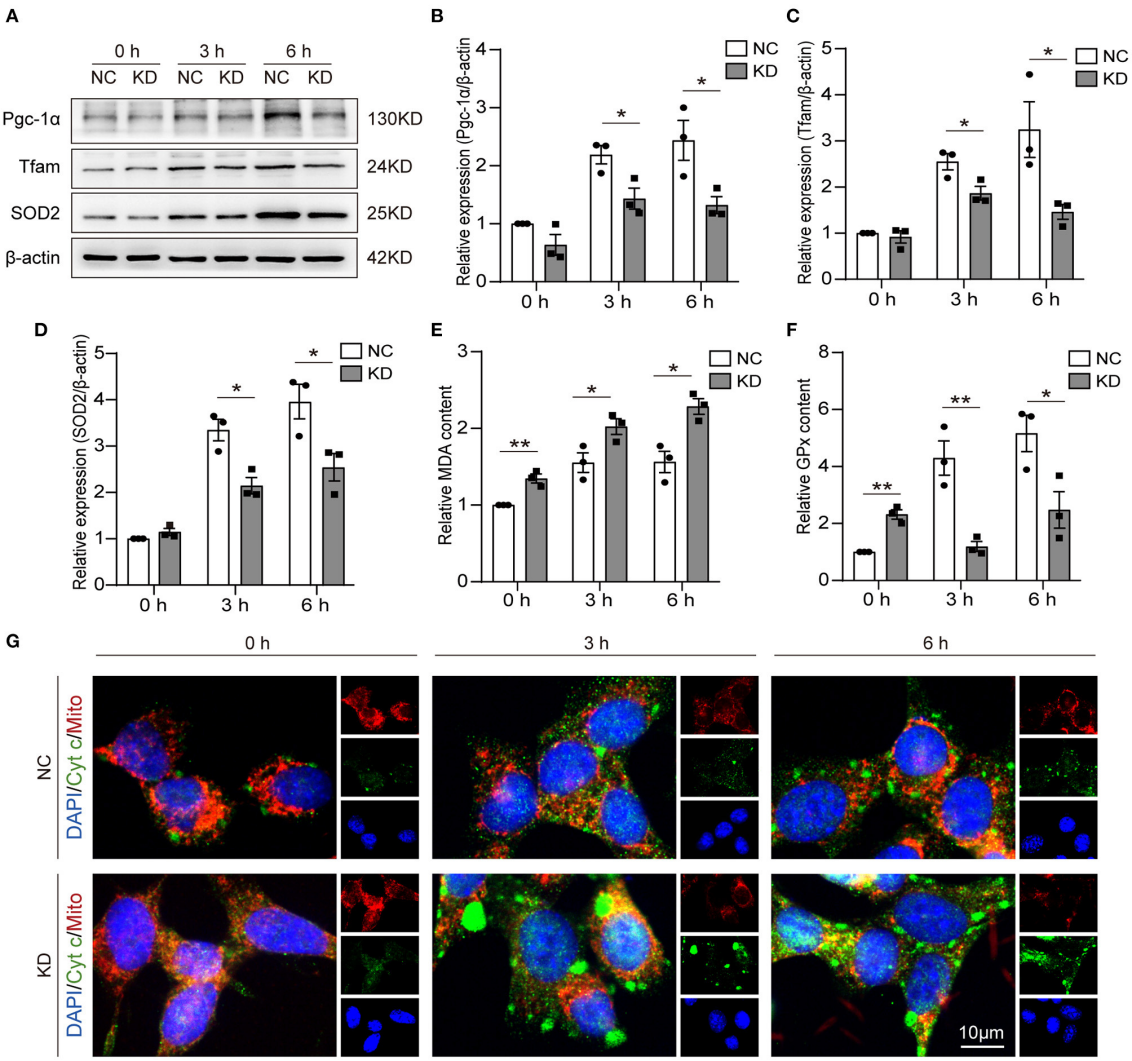
Knockdown of *Fos* aggravated oxidative stress and impaired mitochondrial function. **(A–D)** Representative bands and quantification of SOD2, Pgc-1α, and Tfam from WB. **(E)** MDA levels were assessed in *Fos* knockdown cells after OGD. **(F)** GPx levels were assessed in *Fos* knockdown cells after OGD. **(G)** Representative immunofluorescence microphotographs of the NC and KD groups at each time point after OGD. Mitochondria (Mito) and Cytochrome c (Cyt c) were labeled by red and green puncta, respectively; the nuclear (DAPI) was labeled by blue. Scale bar = 10 μm. Each result is expressed as the mean of three independent experiments ± SEM. ^*^p < 0.05, ^**^p < 0.01. NC, negative control cells; KD, knock-down cells.

One of the mitochondrial biogenesis regulation pathways is the Pgc-1α/Tfam pathway. In our research, we detected the expression of Pcg-1α and Tfam by WB (Figure 5A). Through analysis of the WB results, we found Pgc-1α (Figure 5B) and Tfam (Figure 5C) expression reduced in KD at 3 and 6 h after OGD compared with NC. The MDA, GPx, and SOD2 content can be used to measure antioxidative stress ability. We found that knockdown of *Fos* increased MDA expression and reduced GPx expression at 3 and 6 h after OGD, but MDA and GPx expression was increased in NC at 0 h compared with the KD (Figures 5E,F). SOD2 expression levels were also measured by WB, and the expression level of SOD2 was also reduced in the KD at 3 and 6 h after OGD compared with NC (Figures 5A,D). Through immunofluorescence, we further found that cytochrome c in the cytoplasm of KD was significantly higher than that in NC at 3 and 6 h after OGD, while there was no significant difference in 0 h (Figure 5G). This indirectly implies that *Fos* can attenuate the mitochondrial function of HT22 cells at 3 and 6 h after OGD.

### Knockdown of *Fos* Aggravated Neuronal Apoptosis After Oxygen–Glucose Deprivation

**Figure 6:**
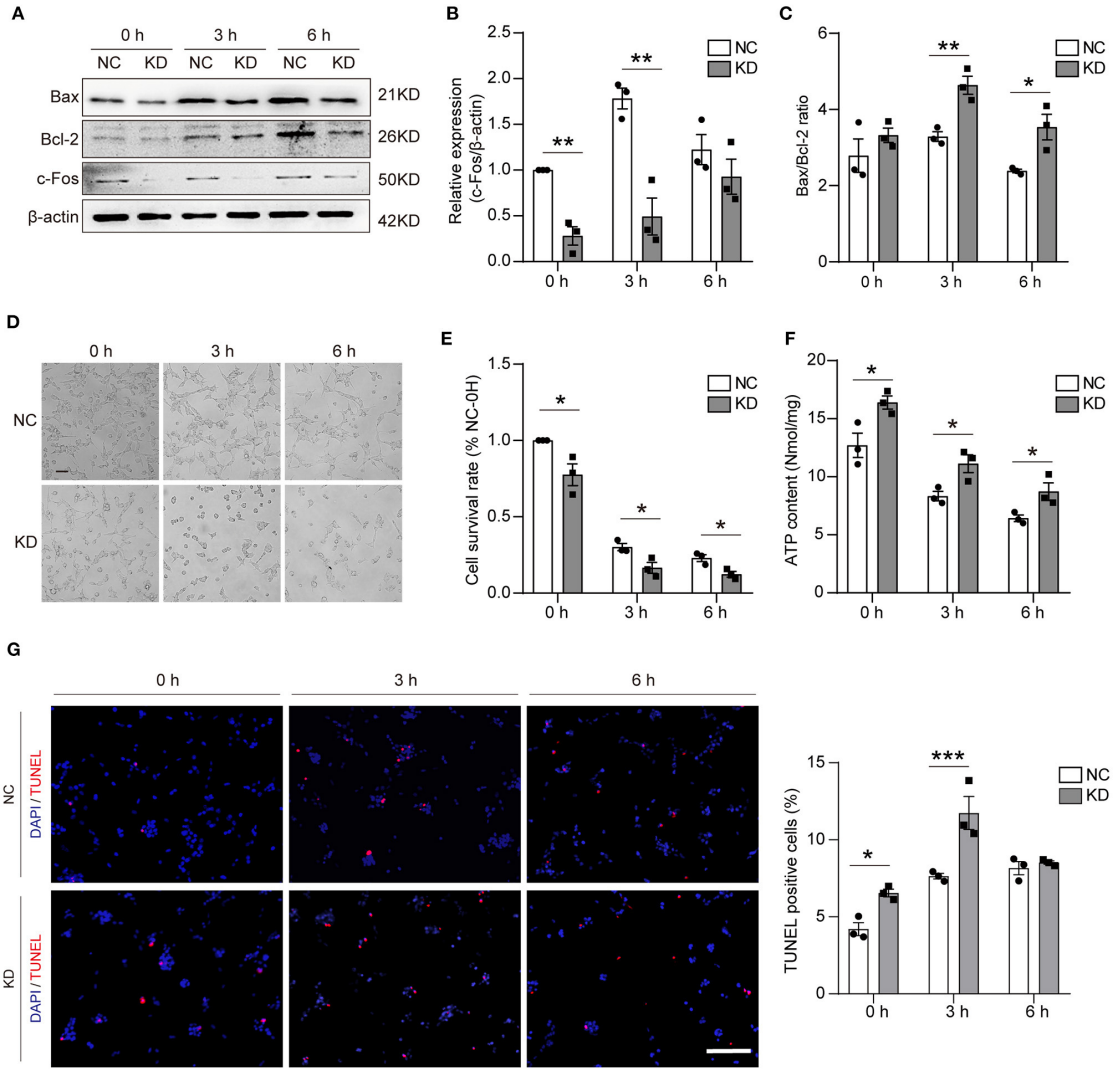
Apoptosis was aggravated in the KD group after OGD. **(A–C)** Representative WB bands and quantification of c-Fos, Bax, and Bcl-2 expression levels. **(D,E)** Cell morphology and cell survival rate at 0, 3, and 6 h. Scale bar = 50 μm. **(F)** ATP content (nmol/mg) was detected by an ATP Assay Kit. **(G)** The TUNEL-positive cells were labed by red, and the nuclear (DAPI) was labeled by blue. Scale bar = 100 μm. Each result is expressed as the mean of three independent experiments ± SEM. ^*^p < 0.05, ^**^p < 0.01, ^***^p < 0.001. NC, negative control cells; KD, knock-down cells.

To assess the effect on apoptosis, different methods were used in our study. First, WB was used to validate the knockdown of *Fos* at different time points after OGD, and the results showed that the knockdown of *Fos* was significant at 0 and 3 h. Knockdown of *Fos* could exacerbate HT22 cell apoptosis by increasing the Bax/Bcl-2 ratio (Figures 6A–C). The number of survived cells of KD was reduced, and cytoplasmic contraction of KD was more apparent at 0, 3, and 6 h after OGD compared with NC. MTT results indicated that the cell survival rate of KD decreased at 0, 3, and 6 h after OGD compared with NC (Figures 6D,E). On the other hand, it also proved that intracellular ATP concentration is an important determinant of cell death. Since mitochondrial structure and function determine ATP production, necrosis is associated with ATP deficiency, while apoptosis required ATP (18). We evaluated the ATP content of our models and found that *Fos* knockdown increased ATP content at 0, 3, and 6 h after OGD (Figure 6F). In addition, the results of TUNEL assay suggested that the number of apoptotic cells was significantly increased in the KD group at 0 and 3 h in HT22 cells with knockdown of *Fos* (Figure 6G), indicating that knockdown of *Fos* in HT22 cells aggravated apoptosis after OGD.

## Discussion

The present study, for the first time, systematically evaluated the transcriptomic profiling interaction between hypertension and IS. Several novel findings were made in the present study: (1) By analyzing microarrays from a variety of stroke samples, we found that *Fos* can act as a key gene between hypertension and stroke. (2) Our high-throughput sequencing data suggest that upregulated genes after stroke are mainly involved in inflammatory and reactive oxygen species (ROS)-related pathways including oxidative stress. (3) *Fos* was increased in neurons at 3 h after OGD, then returned to normal levels at 6 h. (4) Knockdown of *Fos* increased oxidative stress and apoptosis in OGD-treated cells and exacerbated the functional impairment of the mitochondria.

Hypertension has been identified as a key factor associated with the development and prognosis of cerebrovascular disease in patients (especially in older patients) with either ischemic or hemorrhagic strokes. There are several ways in which clinicians can control arterial blood pressure to reduce stroke incidence and manage stroke patients. However, new strategies to identify and reduce stroke risk and improve management of acute stroke are necessary (19).

Notably, pre-stroke hypertension has a substantial impact on the prognostic outcome of IS (20). Pathophysiological processes such as oxidative stress, inflammatory immune response, and structural changes in the vasculature are all involved in the development of stroke or hypertension (21–23). However, exploration of hypertension-related gene expression and their alterations associated with IS has not been reported previously. Therefore, our study on exploring the molecular factors associated with hypertension in pre-stroke and stroke patients might further clarify the impact of hypertension on stroke and, thus, provide theoretical guidance for stroke prevention interventions during the hypertensive phase and pharmacological interventions after stroke.

In this study, gene expression microarray data were extracted from public databases, which included samples of spontaneously hypertensive stroke-prone rats, MCAO rats, peripheral blood of IS patients, and their respective control groups. We identified five hypertension-prone stroke genes and screened for *Fos* with the highest PPI score. Consistent with our findings, Yoshida et al. previously reported that *Fos* is one of the hypertension-prone stroke genes (24). The other four genes are involved in oxidative stress, immune response, and transcriptional regulation, which is consistent with the pathophysiological processes involved in stroke (25–29). For example, ROS can induce the expression of ATF3, which in turn inhibits the activity of the PINK1 promoter and causes accumulation of depolarized mitochondria, increased production of mitochondrial ROS, and loss of cell viability (30, 31). *Fos* is one of the immediate-early genes that are activated transiently and rapidly in response to a wide variety of cellular stimuli. Recent studies revealed that *Fos* plays important roles in cell proliferation, neuron activation, inflammatory, apoptosis, tumor, and oxidative stress (32–35). In addition, our RNA-seq data indicated that (a) *Fos* was increased at 3 h and returned to normal levels at 6 h after OGD compared with control, and (b) the upregulated genes are widely involved in pathophysiological processes such as cellular energy metabolism and oxidative stress.

Hypoxia and oxidative stress have been reported to be closely linked in previous studies. Hypoxia can cause excessive ROS production and, thus, alter redox homeostasis, while both short-term and long-term hypoxic exposure can induce oxidative stress (36, 37). Mitochondria and stroke have become a major topic of research nowadays (38). In our study, by knocking down *Fos*, we found that the GPx and SOD2 levels were significantly reduced, while MDA was significantly increased in OGD-treated neurons. The Pgc-1α/Tfam pathway is downregulated in the KD compared with the NC. Immunofluorescence shows that cytochrome c in the cytoplasm was also significantly increased after *Fos* knockdown. The evidence further suggests that *Fos* may function in regulating oxidative stress and mitochondrial homeostasis during IS. Under oxidative stress, ROS including free radicals such as superoxide, hydroxyl radical, and hydrogen peroxide are generated at high levels inducing cellular damage and cell death. Interestingly, the role of *Fos* in apoptosis appears to be diverse. Some studies suggested that *Fos* can reduce apoptosis, while others suggest that *Fos* can increase apoptosis (34, 39–41). Our study found that knockdown of *Fos* can reduce cell survival rate, increase Bax/Bcl-2 ratio, and increase ATP content, which means knockdown of *Fos* increases neuron apoptosis after OGD. Mitochondria is also one of the new therapeutic targets in stroke, and *Fos* is involved in the regulation of mitochondrial function and oxidative stress, so *Fos* may be a potential new target for stroke therapy (42, 43).

We acknowledge that our results should be interpreted cautiously due to some limitations. The neuronal OGD model is a single-cell model of stroke, a model that helps to control variables and reduce the interference of factors other than neurons. However, the brain has many other types of cells in addition to neurons, and there are multiple cells in the peripheral blood, and stroke is a complex pathophysiological process that can involve many cells. Therefore, the combination of multiple models of cells and animals with each other helps our results to be closer to the reality of the human condition. Further validation in animals or humans in future studies will be necessary to apply our results to clinical practice. Despite many novel findings, we did not provide specific evidence on how *Fos* affects mitochondria and ROS. Further studies are needed to fully establish the underlying mechanism of immediate-early oxidative stress after ischemic injury and the specific role of *Fos* in this process.

## Conclusions

In conclusion, *Fos*, a hypertensive stroke-prone gene, may be involved in the regulation of oxidative stress and neuronal apoptosis after stroke and may represent a new therapeutic target and clinical indicator for stroke.

## Data Availability Statement

The datasets presented in this study can be found in online repositories. The names of the repository/repositories and accession number(s) can be found here: GEO database with the following Accession Number: GSE178997.

## Author Contributions

QM wrote the manuscript and generated the figures. YZ and LG helped to generate and design the figures. SG, XQ, QT, SY, JPa, GD, and LZ proposed suggestions for revisions. JPe revised the manuscript. YJ, JPe, and SY designed the content. All authors have read and approved the final manuscript.

## Funding

This work was supported by grants from the Young Elite Scientist Sponsorship Program by the China Association for Science and Technology and the National Natural Science Foundation of China (81771278, 81801176 and 81971132); the Sichuan Science and Technology Program (2019JDTD0004 and 2019JDRC0062); the doctoral Research Initiation Fund of Affiliated Hospital of Southwest Medical University.

## Conflict of Interest

The authors declare that the research was conducted in the absence of any commercial or financial relationships that could be construed as a potential conflict of interest.

## Publisher's Note

All claims expressed in this article are solely those of the authors and do not necessarily represent those of their affiliated organizations, or those of the publisher, the editors and the reviewers. Any product that may be evaluated in this article, or claim that may be made by its manufacturer, is not guaranteed or endorsed by the publisher.

## References

[1] Muse ED Wineinger NE Spencer EG Peters M Henderson R Zhang Y. Validation of a genetic risk score for atrial fibrillation: a prospective multicenter cohort study. PLoS Med. (2018) 15:e1002525. 10.1371/journal.pmed.1002525 29534064 PMC5849279

[2] Zhao BQ Chauhan AK Canault M Patten IS Yang JJ Dockal M. von Willebrand factor-cleaving protease ADAMTS13 reduces ischemic brain injury in experimental stroke. Blood. (2009) 114:3329–34. 10.1182/blood-2009-03-213264 19687510 PMC2759655

[3] Prenissl J Manne-Goehler J Jaacks LM Prabhakaran D Awasthi A Bischops AC. Hypertension screening, awareness, treatment, and control in India: a nationally representative cross-sectional study among individuals aged 15 to 49 years. PLoS Med. (2019) 16:e1002801. 10.1371/journal.pmed.1002801 31050680 PMC6499417

[4] Fukunaga Y Itoh H Hosoda K Doi K Matsuda J Son C. Altered gene expression of uncoupling protein-2 and−3 in stroke-prone spontaneously hypertensive rats. J Hypertens. (2000) 18:1233–8. 10.1097/00004872-200018090-00009 10994754

[5] Hoffman GE Smith MS Verbalis JG. c-Fos and related immediate early gene products as markers of activity in neuroendocrine systems. Front Neuroendocrinol. (1993) 14:173–213. 10.1006/frne.1993.1006 8349003

[6] Sun Y Lin Z Liu CH Gong Y Liegl R Fredrick TW. Inflammatory signals from photoreceptor modulate pathological retinal angiogenesis *via* c-Fos. J Exp Med. (2017) 214:1753–67. 10.1084/jem.20161645 28465464 PMC5461000

[7] Simpkins AN Janowski M Oz HS Roberts J Bix G Doré S. Biomarker application for precision medicine in stroke. Transl Stroke Res. (2020) 11:615–27. 10.1007/s12975-019-00762-3 31848851 PMC7299765

[8] Watanabe Y Yoshida M Yamanishi K Yamamoto H Okuzaki D Nojima H. Genetic analysis of genes causing hypertension and stroke in spontaneously hypertensive rats: gene expression profiles in the kidneys. Int J Mol Med. (2015) 36:712–24. 10.3892/ijmm.2015.2281 26165378 PMC4533772

[9] Krug T Gabriel JP Taipa R Fonseca BV Domingues-Montanari S FernandezCadenas I. TTC7B emerges as a novel risk factor for ischemic stroke through the convergence of several genome-wide approaches. J Cereb Blood Flow Metab. (2012) 32:1061–72. 10.1038/jcbfm.2012.24 22453632 PMC3367223

[10] Li J Yu H Wang W Fu C Zhang W Han F. Genomic and transcriptomic insights into molecular basis of sexually dimorphic nuptial spines in Leptobrachium leishanense. Nat Commun. (2019) 10:5551. 10.1038/s41467-019-13531-5 31804492 PMC6895153

[11] Kohl M Wiese S Warscheid B. Cytoscape: software for visualization and analysis of biological networks. Methods Mol Biol. (2011) 696:291–303. 10.1007/978-1-60761-987-1_18 21063955

[12] Chin CH Chen SH Wu HH Ho CW Ko MT Lin CY. cytoHubba: identifying hub objects and sub-networks from complex interactome. BMC Syst Biol. (2014) 8(Suppl. 4):S11. 10.1186/1752-0509-8-S4-S11 25521941 PMC4290687

[13] Peng J Wu Y Tian X Pang J Kuai L Cao F. High-throughput sequencing and co-expression network analysis of lncRNAs and mRNAs in early brain injury following experimental subarachnoid haemorrhage. Sci Rep. (2017) 7:46577. 10.1038/srep46577 28417961 PMC5394545

[14] Ratchford AM Esguerra CR Moley KH. Decreased oocyte-granulosa cell gap junction communication and connexin expression in a type 1 diabetic mouse model. Mol Endocrinol. (2008) 22:2643–54. 10.1210/me.2007-0495 18829945 PMC2626198

[15] Frolova A Flessner L Chi M Kim ST Foyouzi-Yousefi N Moley KH. Facilitative glucose transporter type 1 is differentially regulated by progesterone and estrogen in murine and human endometrial stromal cells. Endocrinology. (2009) 150:1512–20. 10.1210/en.2008-1081 18948400 PMC2654750

[16] Helmig S Walter D Putzier J Maxeiner H Wenzel S Schneider J. Oxidative and cytotoxic stress induced by inorganic granular and fibrous particles. Mol Med Rep. (2018) 17:8518–29. 10.3892/mmr.2018.8923 29693699

[17] Luo T Gao J Lin N Wang J. Effects of two kinds of iron nanoparticles as reactive oxygen species inducer and scavenger on the transcriptomic profiles of two human leukemia cells with different stemness. Nanomaterials (Basel). (2020) 10:951. 10.3390/nano10101951 33007950 PMC7600526

[18] Leist M Single B Castoldi AF Kühnle S Nicotera P. Intracellular adenosine triphosphate (ATP) concentration: a switch in the decision between apoptosis and necrosis. J Exp Med. (1997) 185:1481–6. 10.1084/jem.185.8.1481 9126928 PMC2196283

[19] Wajngarten M Silva GS. Hypertension and stroke: update on treatment. Eur Cardiol. (2019) 14:111–5. 10.15420/ecr.2019.11.1 31360232 PMC6659031

[20] Cao Q Zhou S Cai B Wang Q Zhang J Shi R. The impacts of premorbid hypertension treatment on functional outcomes of ischemic stroke. J Neurol Sci. (2016) 363:1–4. 10.1016/j.jns.2016.02.020 27000211

[21] Guzik TJ Touyz RM. Oxidative stress, inflammation, and vascular aging in hypertension. Hypertension. (2017) 70:660–7. 10.1161/HYPERTENSIONAHA.117.07802 28784646

[22] Pistoia F Sacco S Degan D Tiseo C Ornello R Carolei A. Hypertension and stroke: epidemiological aspects and clinical evaluation. High Blood Press Cardiovasc Prev. (2016) 23:9–18. 10.1007/s40292-015-0115-2 26159677

[23] Sun W Ding Z Xu S Su Z Li H. Crosstalk between TLR2 and Sphk1 in microglia in the cerebral ischemia/reperfusion-induced inflammatory response. Int J Mol Med. (2017) 40:1750–8. 10.3892/ijmm.2017.3165 29039449 PMC5716455

[24] Yoshida M Watanabe Y Yamanishi K Yamashita A Yamamoto H Okuzaki D. Analysis of genes causing hypertension and stroke in spontaneously hypertensive rats: gene expression profiles in the brain. Int J Mol Med. (2014) 33:887–96. 10.3892/ijmm.2014.1631 24452243

[25] Onodera Y Teramura T Takehara T Shigi K Fukuda K. Reactive oxygen species induce Cox-2 expression *via* TAK1 activation in synovial fibroblast cells. FEBS Open Bio. (2015) 5:492–501. 10.1016/j.fob.2015.06.001 26110105 PMC4476901

[26] Sontheimer RD Racila E Racila DM. C1q: its functions within the innate and adaptive immune responses and its role in lupus autoimmunity. J Invest Dermatol. (2005) 125:14–23. 10.1111/j.0022-202X.2005.23673.x 15982298

[27] Tanaka T Narazaki M Kishimoto T. IL-6 in inflammation, immunity, and disease. Cold Spring Harb Perspect Biol. (2014) 6:a016295. 10.1101/cshperspect.a016295 25190079 PMC4176007

[28] Woodruff TM Thundyil J Tang SC Sobey CG Taylor SM Arumugam TV. Pathophysiology, treatment, and animal and cellular models of human ischemic stroke. Mol Neurodegener. (2011) 6:11. 10.1186/1750-1326-6-11 21266064 PMC3037909

[29] Zhao J Li X Guo M Yu J Yan C. The common stress responsive transcription factor ATF3 binds genomic sites enriched with p300 and H3K27ac for transcriptional regulation. BMC Genomics. (2016) 17:335. 10.1186/s12864-016-2664-8 27146783 PMC4857411

[30] Bueno M Brands J Voltz L Fiedler K Mays B St Croix C. ATF3 represses PINK1 gene transcription in lung epithelial cells to control mitochondrial homeostasis. Aging Cell. (2018) 17:720. 10.1111/acel.12720 29363258 PMC5847866

[31] Hoetzenecker W Echtenacher B Guenova E Hoetzenecker K Woelbing F Brück J. ROS-induced ATF3 causes susceptibility to secondary infections during sepsis-associated immunosuppression. Nat Med 18(1). (2011) 128–34. 10.1038/nm.2557 22179317 PMC3555699

[32] Hsieh HL Wang HH Wu CY Yang CM. Reactive oxygen species-dependent c-Fos/activator protein 1 induction upregulates heme oxygenase-1 expression by bradykinin in brain astrocytes. Antioxid Redox Signal. (2010) 13:1829–44. 10.1089/ars.2009.2957 20486760

[33] Motrich RD Castro GM Caputto BL. Old players with a newly defined function: Fra-1 and c-Fos support growth of human malignant breast tumors by activating membrane biogenesis at the cytoplasm. PLoS ONE. (2013) 8:e53211. 10.1371/journal.pone.0053211 23301044 PMC3534677

[34] Preston GA Lyon TT Yin Y Lang JE Solomon G Annab L. Induction of apoptosis by c-Fos protein. Mol Cell Biol. (1996) 16:211–8. 10.1128/MCB.16.1.211 8524298 PMC230994

[35] Wagner EF Eferl R. Fos/AP-1 proteins in bone and the immune system. Immunol Rev. (2005) 208:126–40. 10.1111/j.0105-2896.2005.00332.x 16313345

[36] Dosek A Ohno H Acs Z Taylor AW Radak Z. High altitude and oxidative stress. Respir Physiol Neurobiol. (2007) 158:128–31. 10.1016/j.resp.2007.03.013 17482529

[37] Joanny P Steinberg J Robach P Richalet JP Gortan C Gardette B. Operation Everest III (Comex'97): the effect of simulated sever hypobaric hypoxia on lipid peroxidation and antioxidant defence systems in human blood at rest and after maximal exercise. Resuscitation. (2001) 49:307–14. 10.1016/S0300-9572(00)00373-7 11723998

[38] Hayakawa K Esposito E Wang X Terasaki Y Liu Y Xing C. Transfer of mitochondria from astrocytes to neurons after stroke. Nature. (2016) 535:551–5. 10.1038/nature18928 27466127 PMC4968589

[39] Pirzad Jahromi G P Shabanzadeh A Mokhtari Hashtjini M Sadr SS Rasouli Vani J Raouf Sarshoori J. Bone marrow-derived mesenchymal stem cell and simvastatin treatment leads to improved functional recovery and modified c-Fos expression levels in the brain following ischemic stroke Iran. J Basic Med Sci. (2018) 21:1004–12. 10.22038/IJBMS.2018.29382.7100 30524673 PMC6281073

[40] Yuan Z Gong S Luo J Zheng Z Song B Ma S. Opposing roles for ATF2 and c-Fos in c-Jun-mediated neuronal apoptosis. Mol Cell Biol. (2009) 29:2431–42. 10.1128/MCB.01344-08 19255142 PMC2668374

[41] LZ Man QW Liu JY Zhong WQ Zheng YY Zhao YF. Overexpression of Fra-1, c-Jun and c-Fos in odontogenic keratocysts: potential correlation with proliferative and anti-apoptotic activity. Histopathology. (2018) 73:933–42. 10.1111/his.13705 29993138

[42] Chen W Huang J Hu Y Khoshnam SE Sarkaki A. Mitochondrial transfer as a therapeutic strategy against ischemic stroke. Transl Stroke Res. (2020) 11:1214–28. 10.1007/s12975-020-00828-7 32592024

[43] Park JH Lo EH Hayakawa K. Endoplasmic reticulum interaction supports energy production and redox homeostasis in mitochondria released from astrocytes. Transl Stroke Res. (2021). 10.1007/s12975-021-00892-7 33479917 PMC8324082

