# Supplementary material for: Transcriptomic Profiling Reveals the Antiapoptosis and Antioxidant Stress Effects of Fos in Ischemic Stroke
Source: Front Neurol. 2021 Oct 21;12:728984. doi: 10.3389/fneur.2021.728984 (PMC8566985; doi:10.3389/fneur.2021.728984)
Supplement: Supplementary file 1 [file Data_Sheet_1.docx]

# Transcriptomic profiling reveals the anti-apoptosis and antioxidant stress effects of *Fos* in ischemic stroke

**Qiancheng Mu****^1,2‡^, Yuxuan Zhang^1,2‡^, Long Gu^2^, Stefan T. Gerner^3^, Xiancheng Qiu^1,2^, Qianke Tao^1,2^, Jinwei Pang^1,2,4,5^, Ghosh Dipritu^1,2^, Lifang Zhang^2,6^, Shigang Yin^2,4,5^, Yong Jiang^1,2,4,5^* and Jianhua Peng^1,2,5,6^***

^1^Department of Neurosurgery, the Affiliated Hospital of Southwest Medical University, Luzhou 646000, China;

^2^Luzhou Key Laboratory of Neurological Diseases and Brain Function, the Affiliated Hospital of Southwest Medical University, Luzhou 646000, China;

^3^Department of Neurology, University Hospital Erlangen-Nuremberg, Erlangen 91054, Germany.

^4^Academician (Expert) Workstation of Sichuan Province, the Affiliated Hospital of Southwest Medical University, Luzhou 646000, China;

^5^Institute of Epigenetics and Brain Science, Southwest Medical University, Luzhou 646000, China

^6^Sichuan Clinical Research Center for Neurosurgery, the Affiliated Hospital of Southwest Medical University, Luzhou 646000, China;

^‡^ These authors contributed equally to this work.

*** Correspondence:**

Jianhua Peng

[pengjianhua@swmu.edu.cn](mailto:pengjianhua@swmu.edu.cn)

Yong Jiang

[jiangyong@swmu.edu.cn](mailto:jiangyong@swmu.edu.cn)

**Supplementary materials**

Supplement Fig. S1. Cluster analysis of the samples.

Supplement Fig. S2. Selection of the best soft threshold and module.

Supplement Fig. S3. Volcano plots and GO analysis of up-regulated genes for RNA-seq data from OGD-treated cells.

Supplement Fig. S4. KEGG pathway enrichment analysis was used for RNA-seq data.

Supplement Table S1. Differentially expressed genes in peripheral blood of ischemic stroke patients.

Supplement Table S2. Characterization of the control individuals and stroke patients.


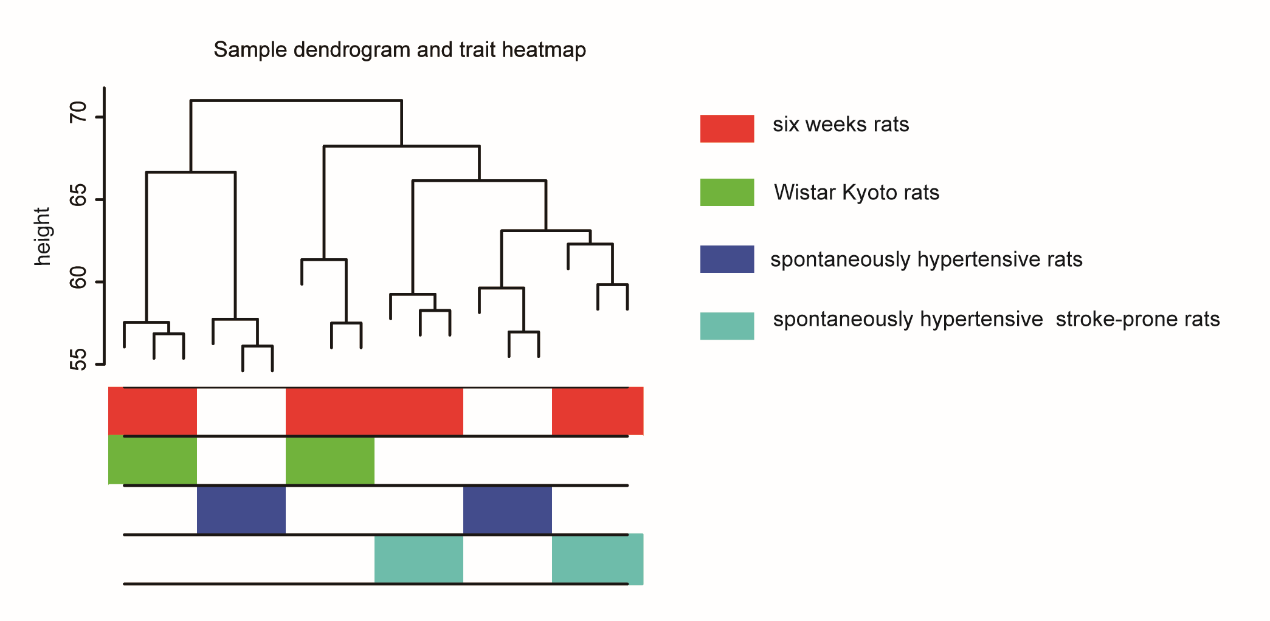


**Fig. S1. Cluster analysis of the samples.** The upper part shows the relationship between the samples. The similar samples were preferentially converged under the same branch, and the ordinate was the distance between the samples. The lower part shows the sample distribution corresponding to the upper part of the tree clustering. The red indicates that the age of the rat was 6 weeks and 3 weeks was white; the green indicates that the rat was the control group, the rest was white; the blue indicates that the rat was a non-stroke-prone hypertensive rat, and the rest was white. The emerald green indicates that the rats were hypertensive and prone to stroke, while the others were white.


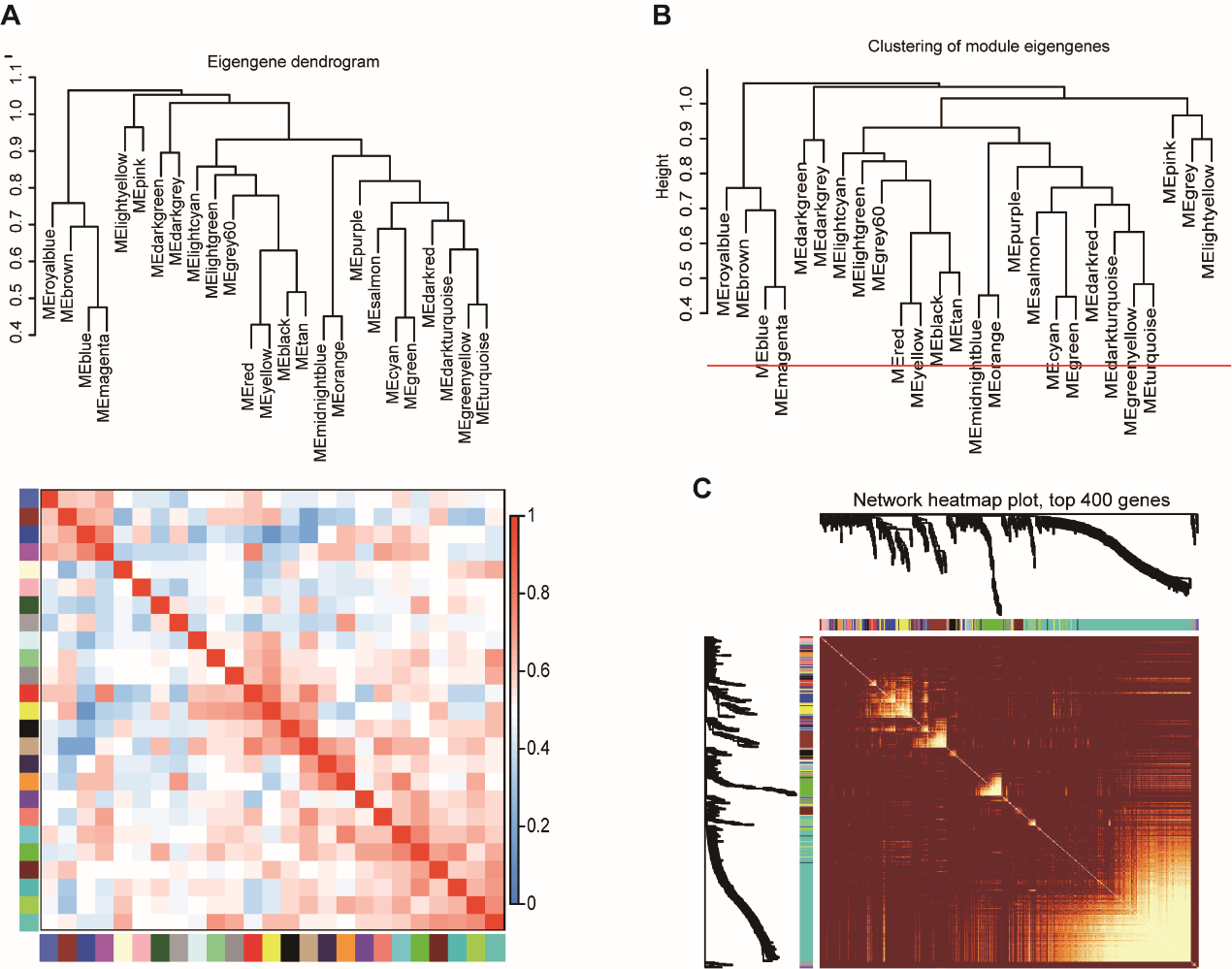


**Fig. S2. Selection of the best soft threshold and module.** (A) The tree clustering relationship between modules is shown, the lower part shows the similarity of gene principal components in the module, different colors represent the corresponding module, the closer to 1 means the greater the similarity, and the closer to 0 means the smaller the similarity. (B) Clustering of module eigengenes is shown, and the red line represents a position with a height of 0.25. (C) Network heatmap plot of the top 400 genes produced by correlation analysis. Highlight indicates that there was a correlation.


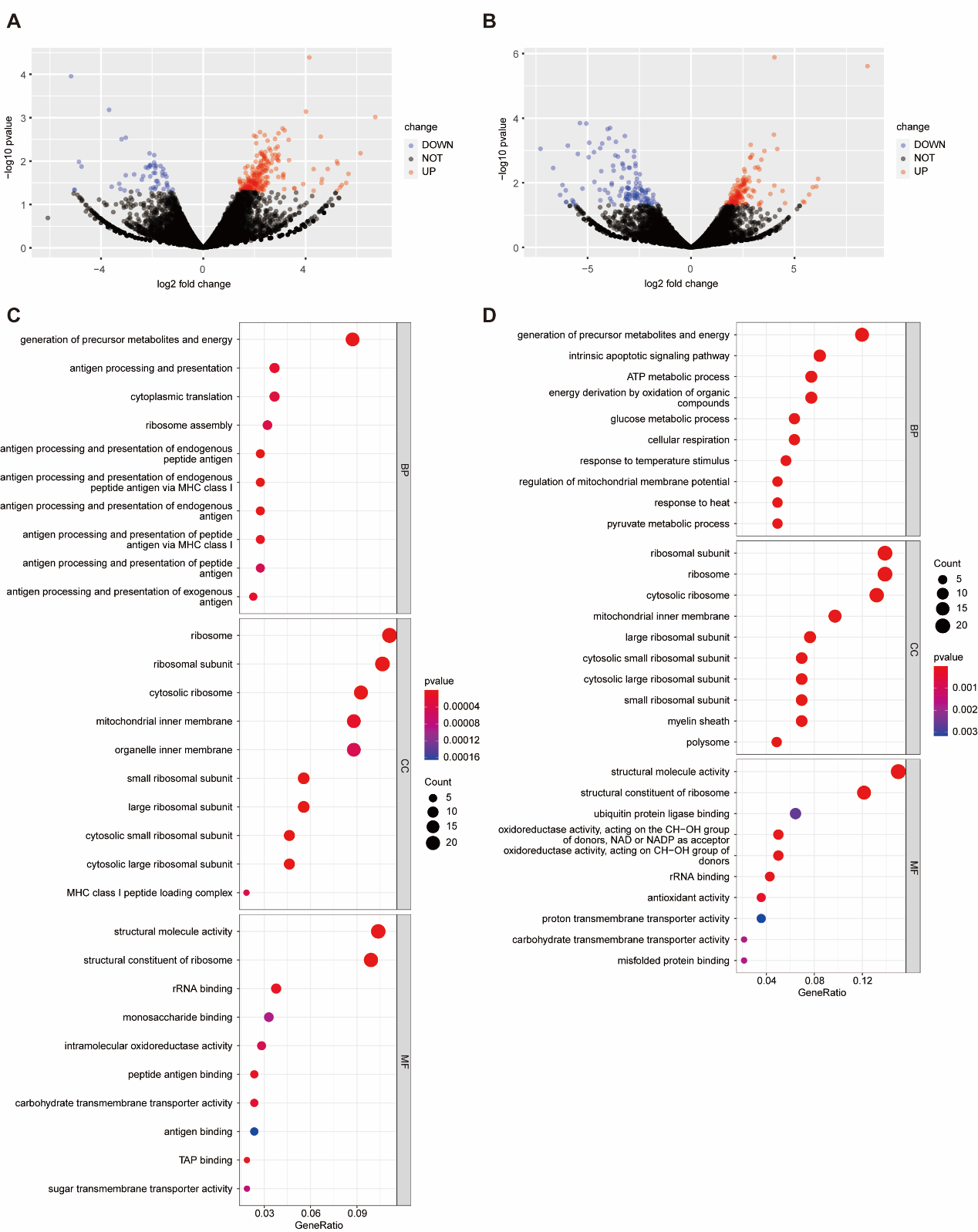


**Fig. S3. Volcano plots and GO analysis of up-regulated genes for RNA-seq data from OGD-treated cells.** Differentially expressed RNAs were filtered using the criteria of log_2_FC = 1.5 and corrected *p*-value <0.05 using the R software package. These criteria identified 229 up-regulated and 50 down-regulated genes in 6 h-vs-3 h (A), and 145 up-regulated and 121 down-regulated genes in 6 h-vs-0 h (B), in which the red and green spots represent upregulated and downregulated differentially expressed genes (DEGs), respectively. GO enrichment analysis of the up-regulated DEGs in RNA-seq data of 6 h vs 3 h (C) and 6 h vs 0 h (D). Gene Ratio indicates the number of genes enriched in one pathway compared with the total genes changed in all pathways. Count indicates the number of genes. The color represents *p-*value and red is the highest. BP, biological process; CC, cellular component; MF, molecular function.


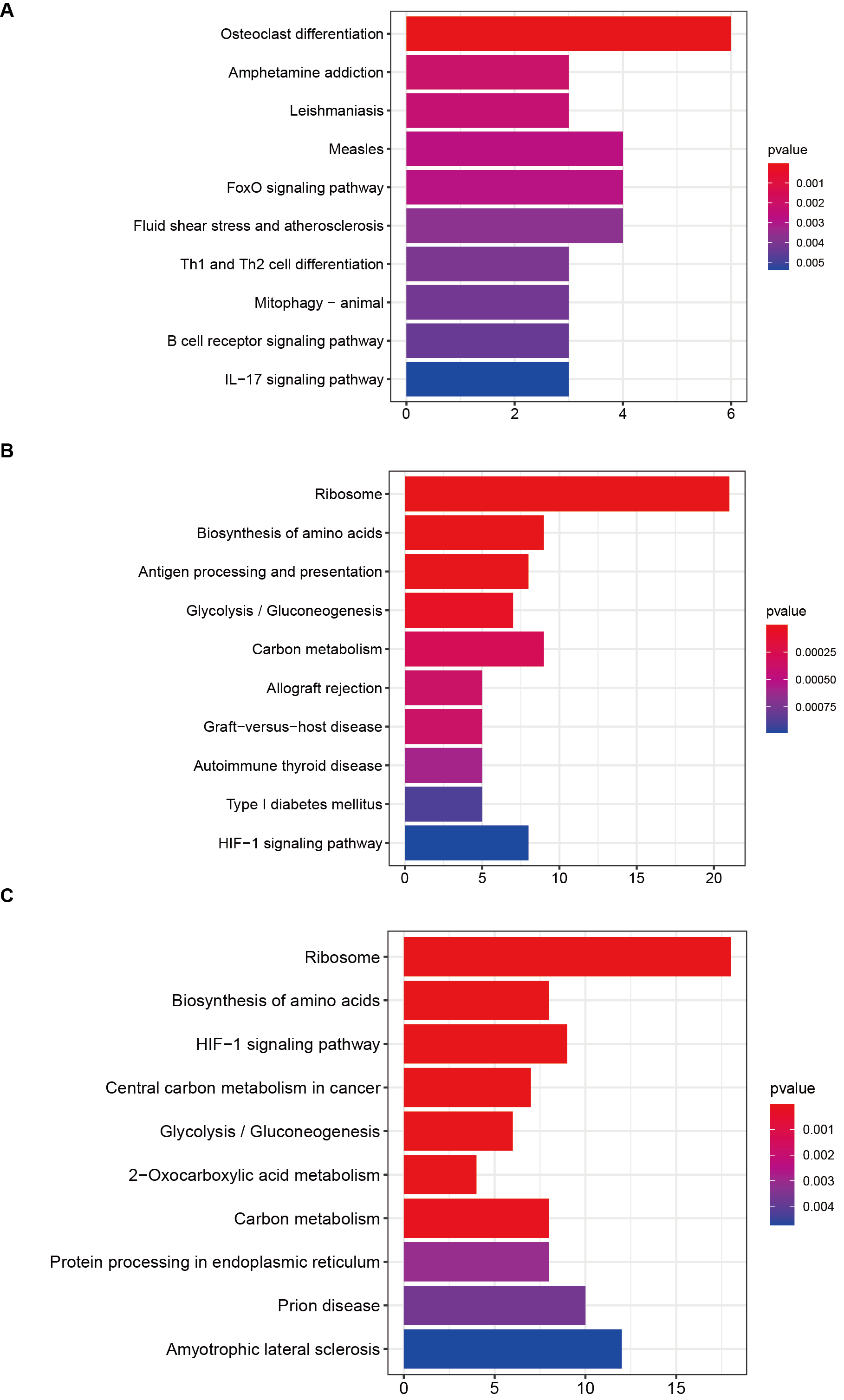


**Fig. S4. KEGG pathway enrichment analysis was used for RNA-seq data.** R package clusterProfiler was used for KEGG pathway enrichment analysis of DEGs in the screened key modules, which were divided into 3 h-vs-0 h (A), 6 h-vs-3 h (B) and 6 h-vs-0 h (C). Fisher exact probability test was the statistical method. Horizontal coordinates represent the number of genes, vertical coordinates represent signaling pathways, colors represent *p*-value and red is the lowest.

**Table S1. Differentially expressed genes in peripheral blood of IS patients.**

| Symbol | LogFC | *P*-Value | change |
| --- | --- | --- | --- |
| JUN | 1.976481 | 0.000993 | UP |
| LOC101928152 | 1.240694 | 0.002444 | UP |
| CXCL8 | 3.19411 | 0.002832 | UP |
| ATP2B1-AS1 | 1.308176 | 0.005247 | UP |
| TNF | 2.311721 | 0.00545 | UP |
| G0S2 | 2.868647 | 0.005649 | UP |
| EGR1 | 2.172899 | 0.006072 | UP |
| IER3 | 1.420678 | 0.006154 | UP |
| OSM | 1.716475 | 0.006225 | UP |
| LOC399900 | 1.125508 | 0.006504 | UP |
| NFKBIA | 1.003723 | 0.009801 | UP |
| TNFAIP3 | 1.1447 | 0.010629 | UP |
| CD69 | 1.122154 | 0.011138 | UP |
| DUSP2 | 1.566959 | 0.014492 | UP |
| PPP1R15A | 1.397673 | 0.016061 | UP |
| FOS | 1.100396 | 0.019536 | UP |
| CXCL2 | 2.838493 | 0.021906 | UP |
| PTGS2 | 2.162752 | 0.022172 | UP |
| TRBV27 | 1.157311 | 0.028142 | UP |
| RGS1 | 1.675769 | 0.028718 | UP |
| SOCS3 | 1.232953 | 0.030634 | UP |
| ATF3 | 1.409885 | 0.034875 | UP |
| NR4A2 | 1.958103 | 0.043755 | UP |
| IL1B | 2.000295 | 0.045036 | UP |
| BABAM2-AS1 | 1.795195 | 0.045811 | UP |
| CD83 | 1.624993 | 0.046379 | UP |
| FOSB | 1.628647 | 0.048365 | UP |
| CLEC4C | -1.02712 | 0.000363 | DOWN |
| EIF5A | -1.61829 | 0.002865 | DOWN |
| TNFRSF17 | -1.35095 | 0.005725 | DOWN |

**Table S2: Characterization of the control individuals and stroke patients**

| **Samples used in the gene profiling (n = 40)** | **Control**  **(n = 20)** | **Ischemic stroke**  **(n = 20)** | ***P*-Value** |
| --- | --- | --- | --- |
| Age-at-examination, median (inter-quartile range) | 59 (48-68.75) | 59.5 (49.75-70.75) | 0.432 |
| Age-at-onset, median (inter-quartile range) | - | 59 (47.25-65.75) | - |
| Female sex, No. (%) | 10 (50%) | 10 (50%) | >0.999 |
| Hypertension, No. (%) | 8 (40%) | 12 (60%) | 0.343 |
| Diabetes, No. (%) | 0 (0.0%) | 4 (20%) | 0.106 |
| Hypercholesterolemia, No. (%) | 7 (35%) | 10 50%) | 0.523 |
| Ever drinker, No. (%) | 8 (40%) | 13 (65%) | 0.204 |
| Ever smoker, No. (%) | 6 (30%) | 4 (20%) | 0.716 |

Data was derived from the research by Krug et al. (*J Cereb Blood Flow Metab, 2012,* 32(6), 1061-1072.)
